# Improving patient safety through medicine risk communication: a theory-informed evaluation approach

**DOI:** 10.1108/IJHCQA-12-2025-0218

**Published:** 2026-05-29

**Authors:** Anna V. Chatzi

**Affiliations:** University of Limerick, Limerick, Ireland

**Keywords:** Medication risk communications, Behavioural change, Healthcare practitioners, Patient safety, Human factors

## Abstract

**Purpose:**

The growing population and consequent augmented medicinal needs place an emphasis on effective pharmacovigilance processes. Patient safety may be optimised by further managing risks to prevent adverse drug reactions, which can be associated with significant hospital admissions and proportionate relevant monetary expenditure.

**Design/methodology/approach:**

In this rapidly changing landscape, under the pressing demand to enhance patient safety, this conceptual paper offers an evidence-based perspective on the need and type of evaluation of the systems used for medicine risk communications.

**Findings:**

National health products and medicines regulatory bodies/authorities are using approved outlets and distribution modes to communicate medicines safety issues timely and efficiently to healthcare practitioners. However, medicine risk communications need to be periodically evaluated on their effectiveness in achieving the desired patient outcomes regarding the acceptance and adherence by the targeted healthcare professionals.

**Originality/value:**

The novelty of this study lies in its integration of behavioural science and human factors into the evaluation of medicine risk communication systems, offering a conceptual framework that moves beyond process metrics to include behavioural outcomes critical for patient safety.

## Introduction

Healthcare systems are well recognised for their highly regulatory nature with complicated and multilayered supporting/bureaucratic processes aiming at patients' safety. However, literature has identified patient harm to continue to be consistently high (Back *et al*., 2024; World Health Organization, 2023). The World Health Organisation (WHO) has revealed that healthcare exhibits worse safety records with higher risk levels when compared to other critical industries such as aviation and nuclear (World Health Organization, 2019). Among all healthcare activities and areas, unsafe medication practices and medication errors have been linked globally with patients/clients' damages and preventable harm. The latest relevant monetary global cost is in the range of 42 billion dollars annually, a fraction of 1% of the total global healthcare expenditure, with the WHO stressing the need for an immediate action plan (Durand *et al*., 2024). In line with enhancing patient safety, the use of systematic approaches has revealed the need for design improvements of multiple system structures and processes (World Health Organization, 2023).

Within medication management, pharmacovigilance is a framework that aims to identify and manage potential safety issues of medicinal products that have been authorised for use. Within healthcare's wider evolving landscape (i.e. the need to address unmet medical needs, public health emergencies, to encourage innovation, to improve patient access and augment the impact of global health in total), the expedited approval processes for medicinal products have been deemed necessary. Expedited approval processes are there to support the public's access to medicines with a positive benefit/risk balance. At the same time, they do bring into focus the importance of effective post-market lifecycle management and the need for integration of the outcomes from pharmacovigilance in clinical practice. From observations in different jurisdictions, the increased speed of these processes could potentially be associated with a higher number of warnings and alerts at the post-marketing phase (Bhasale *et al*., 2021).

Pharmacovigilance plays a critical role in medication management by offering a systematic framework to detect and address potential safety issues associated with authorised medicines. Regulatory systems worldwide incorporate mandatory risk minimisation strategies as a fundamental component, ensuring medicines are used appropriately and patient benefits are maintained through effective risk management (Nyeland *et al*., 2017; Perry *et al*., 2025). In pharmacovigilance, risk minimisation measures (RMMs) are regulatory strategies designed to ensure that a medicine's benefits continue to outweigh its risks during real-world use. Standard or routine RMMs apply to all approved medicines and typically include tools such as the Summary of Product Characteristics, patient information leaflets, product labelling and ongoing safety monitoring. When these routine measures are insufficient to address specific safety concerns, authorities may implement additional RMMs. These targeted actions can include direct healthcare professional communications (DHPCs), prescribing or dispensing restrictions, pregnancy prevention programs (PPP) and educational resources for healthcare providers or patients. Such measures aim to guide clinical decisions, enhance safe use and support informed practice.

This dynamic and challenging landscape poses the need for consistent evaluation of effectiveness (Nyeland *et al*., 2017). Various frameworks and models have been introduced to assess how well RMMs are implemented. A key component of these strategies involves ensuring that relevant safety information is communicated to prescribers. Although these models differ in design and methodology, they uniformly emphasise the need to assess the communication aspect, particularly its influence on prescribers' knowledge and behaviour, since patient safety ultimately depends on how healthcare professionals incorporate this information into their clinical practice (Nyeland *et al*., 2017; Perry *et al*., 2025; Alharbi *et al*., 2023). There is still no consistent, standardised approach for routinely evaluating the effectiveness of risk minimization measures (Ahlqvist Rastad *et al*., 2016). However, the importance of employing systematic and rigorous research methods to assess medication safety communications is well recognised (Alharbi *et al*., 2023; Bhasale *et al*., 2021; Weatherburn *et al*., 2020; Møllebæk *et al*., 2019).

Despite the existence of multiple frameworks for evaluating RMMs, prior studies reveal notable inconsistencies and methodological limitations. For instance, surveys across European countries report mixed preferences for communication formats and variable awareness of DHPCs (de Vries *et al*., 2017; Hughes *et al*., 2021), while UK-based research highlights a persistent gap between awareness and implementation of safety updates (Hitchings *et al*., 2013). These findings suggest that dissemination alone does not guarantee behavioural change. Furthermore, many studies rely on small samples and lack inferential analysis, limiting generalizability (Hughes *et al*., 2021). There is also debate regarding the influence of behavioural factors—such as trust in the sender and habitual clinical routines—on the uptake of safety communications (Møllebæk and Kaae, 2021). Collectively, these gaps underscore the need for systematic, theory-informed evaluations that address both process metrics and behavioural outcomes.

## Aim

The aim of this conceptual paper is to offer an evidence-based perspective on the need of integrating behavioural science and human factors into the evaluation of medicine risk communications. These medicine risk communications are an integral part of the regulatory framework for the safe use of medicinal products and, consequently, a big contributor to patient safety.

## Medicine risk communication

There have been several models and frameworks proposed in the evaluation of the used RMMs. Communicating any relevant information to prescribers falls within the wider system of RMMs. Among the different processes and layouts, the models agree that the evaluation of the medicines' safety communications component is a fundamental part, particularly when targeting the prescribers' knowledge and behaviour (Nyeland *et al*., 2017).

Within the European Union (EU), major national safety events have served as focal points of change, with the introduction of directives and regulations, aiming to augment transparency and patient trust, with the ultimate goal to strengthen pharmacovigilance (Chemtob, 2025). In 2012, major changes were implemented, which allowed the European Medicines Agency (EMA) and all EU National Competent Authorities to work together in a structured and legally binding pharmacovigilance system (Santoro *et al*., 2017). Through this new system, EU member states are using many different formats and means to communicate safety information and appear very active in facilitating the communication process (Ahlqvist Rastad *et al*., 2016; Bhasale *et al*., 2021).

For example, in Ireland, the DHPC is approved by the Health Product Regulatory Authority|Health Products Regulatory Authority (HPRA) and distributed by the pharmaceutical companies to all relevant registered doctors and pharmacists. The detailed process from the identification of the need for the creation of the DHPC to its receipt by Health Care Professionals (HCPs) is included in Figure 1. In Ireland, the distribution of the DHPC is done by both email and post, while it is also uploaded and accessible through the HPRA website (de Vries *et al*., 2017). In addition to the DHPC, the HPRA communicates medication safety information by disseminating a safety newsletter by email and by publishing it online on HPRA's website (de Vries *et al*., 2017). Also, the same information is communicated through the press release of the EMA and HPRA's own press release, which are again published online on HPRA's website (de Vries *et al*., 2017).

**Figure 1:**
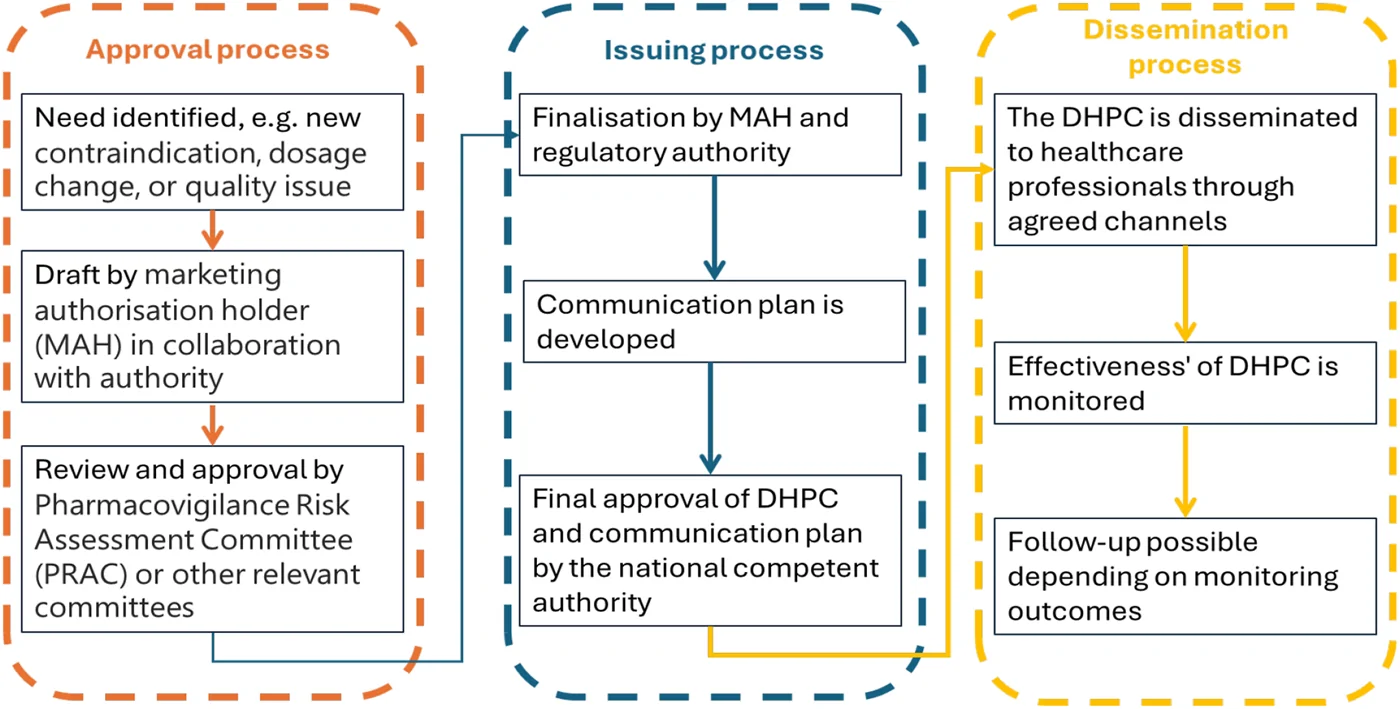
Approval, issuing and dissemination process for DHPC

## The behavioural aspect in medicine risk communications

Risk communication in pharmacovigilance serves a dual function: it is primarily a regulatory activity mandated to ensure timely dissemination of safety information, but it also operates as a behavioural intervention aimed at influencing healthcare professionals' decisions and actions. While regulatory frameworks emphasise compliance and legal obligations, the behavioural perspective focuses on how prescribers interpret, trust and integrate these messages into clinical practice. Conceptualising risk communication through both lenses is essential, as effectiveness depends not only on message delivery but also on its capacity to drive behavioural change.

Exploring risk and safety communications, especially under the prism of behaviour and perception research, has been discussed and supported by the research literature (Bhasale *et al*., 2021; Møllebæk and Kaae, 2022; Nyeland *et al*., 2017) and by EMA. The HCPs' behavioural indicators affect their alignment with the recommended actions communicated through risk messages (Nyeland *et al*., 2017); these behavioural changes serve the overarching goal of protecting patients by supporting the safe and appropriate use of medicines in clinical practice. Similarly, the HCPs' knowledge indicators also affect their awareness and knowledge on the medicinal information that the risk communication intends to pass on (Nyeland *et al*., 2017).

To understand better HCPs' behaviours towards medicine risk communications, it is important to examine them within the frame of their behaviour towards adherence on clinical practice guidelines in general. Medicine risk communications do not exist in isolation as clinicians are called to adjust and adopt multiple clinical practice guidelines while taking personal responsibility for them. The nature of these clinical guidelines themselves has been identified as a barrier to implementation, for being regarded as difficult to follow due to inconsistencies and a lack of uniformity (Kastner *et al*., 2015; Vaghi *et al*., 2025; Carlsen and Bringedal, 2011). Therefore, the need for clear and consistent language in any communication material that reaches HCPs has been established.

Also, other very important barriers are that many clinicians feel their judgment is above the communicated/new clinical practice guidelines (Carlsen and Bringedal, 2011; Barth *et al*., 2016; Lugtenberg *et al*., 2011). This is an indication that they do not easily adapt to new knowledge (Wiener-Ogilvie *et al*., 2007) as it is challenging for them to change their already established clinical routines (Barth *et al*., 2016; Lugtenberg *et al*., 2011). Even though research indicates that the new generation of clinicians work better in teams and appear to adhere to guidelines better (Barth *et al*., 2016), recent literature links these barriers with many patients still not receiving appropriate treatment (Khalaf *et al*., 2024).

Within the remit of strengthening patient safety, HCPs' behavioural change is among the factors that are expected to lead to safer healthcare. Education and training are among the vehicles used for behavioural change; however, inclinations and certain habits need to be considered and catered for (Heinström, 2003; Kil *et al*., 2024). Human factors is the area of research that explores human capabilities and limitations, as it helps humans understand their place and contribution within a working system. Defined as “*the environmental, organizational and job-related factors, along with human and individual characteristics, which impact on workplace behaviour and potentially health and safety*” (O'Connor and O'Dea, 2021), the knowledge of human factors, such as communication, trust, knowledge, motivation, teamwork, biases, etc. Can greatly impact clinical performance, therefore, patient safety. The WHO has already recognised the need for HCPs' uniform knowledge and training in human factors, as an enabler in achieving patient safety (World Health Organization, 2011).

Within the broad domain of human factors, this paper prioritises constructs most directly influencing healthcare professionals' interpretation and application of risk communications. Specifically, trust in the information source, cognitive workload and habitual clinical routines are emphasised because they have been repeatedly identified as barriers to guideline adherence and safety communication uptake (Carlsen and Bringedal, 2011; Møllebæk and Kaae, 2021). Motivation, both reflective and automatic, alongside communication clarity, are also central, as they underpin prescribers' willingness and ability to integrate safety messages into practice. These constructs were selected based on their prominence in prior empirical studies and their alignment with established behaviour-change frameworks (COM-B, EAST and Health Belief Model), ensuring theoretical coherence and practical relevance.

## The role of research in evaluating medication safety communications

The use of rigorous research methods in evaluating the medication safety communications has been acknowledged (Bhasale *et al*., 2021). In the United Kingdom (UK) a wide consultation took place in 2022 among HCPs, relevant organisations, representatives and led to identifying needs for improvement to the existing risk communications. This action falls under the movement to strengthen patient safety and support the healthcare community by improving their critical medicines communications further (Medicines and Healthcare Products Regulatory Authority, 2024).

However, a standardised and periodical monitoring system of the process, which would enable its assessment of its effectiveness, is missing (Ahlqvist Rastad *et al*., 2016). In a recent European survey study, general practitioners' feedback (*N* = 1766), from 26 European countries, has been sought on their preferred distribution means of DHPC and relevant safety medicines communications (de Vries *et al*., 2017). Results were mixed among all participants, with Irish GPs (*N* = 144) equally preferring electronic and hard copy formats, with the majority of participants acknowledging the importance and efficiency of recurrent safety communication (de Vries *et al*., 2017). In another study, when asked on the amount of information they received in regards to the Pregnancy Prevention Programme (PPP) of medicine “Epilim”, Irish GPs (*N* = 59), pharmacists (*N* = 79) and specialist doctors (*N* = 10) gave mixed responses (Hughes *et al*., 2021). The results of these studies, with limited sample sizes, did not include inferential statistical analysis for generalisation of results.

In another European survey, Irish GPs, cardiologists and pharmacists were included (among eight other European countries' clinicians, *N* = 3,288) (de Vries *et al*., 2018). This survey was on participants' understanding of the general purpose of the DHPC and their knowledge on recent safety communications, on certain medicines that had been communicated through DHPC. Results showed that HCPs had a good general understanding of the existence and purpose of DHPC (de Vries *et al*., 2018). However, results indicated that critical information in the area of specialisation does not reach the specialist doctors when this information is for medicines outside their immediate therapeutic area (de Vries *et al*., 2018).

In the UK, doctors, nurses and pharmacists were surveyed (*N* = 267) on their awareness and implementation, twelve weeks after they had received changes of one medication's license terms (Hitchings *et al*., 2013). These changes were communicated through the Medicines and Healthcare Products Regulatory Agency (MHRA) website alert and relevant changes to the British National Formulary (from the 64th edition, September 2012) (Hitchings *et al*., 2013). Results showed that less than two-thirds of the practitioners were aware of these changes, while only half appeared to have implemented the changes into their everyday practice (Hitchings *et al*., 2013). Looking closely into the many different ways used for the risk communication to have reached these practitioners, the smallest proportion belongs to the DHPC (1%), less than one-third were informed through the regulator and three-quarters through their employer's internal alert or other professional sources. Also, this project identified a gap between the proportion of clinicians who were aware of the change and those who have actually implemented the change (Hitchings *et al*., 2013). These findings support establishing an evaluation pillar on process/reach metrics. Conducting timely and relevant surveys with robust statistical analysis and quick turnaround for timely publication are important metrics of the process and reach of the risk communications (Pillar 1, Figure 2). Furthermore, a second pillar on the captured context is necessary. Traits such as trust in sender, workload, local systems and culture of guideline adherence can provide insights into contextual conditions under which HCPs receive, interpret and act on safety messages (Pillar 2, Figure 2).

**Figure 2:**
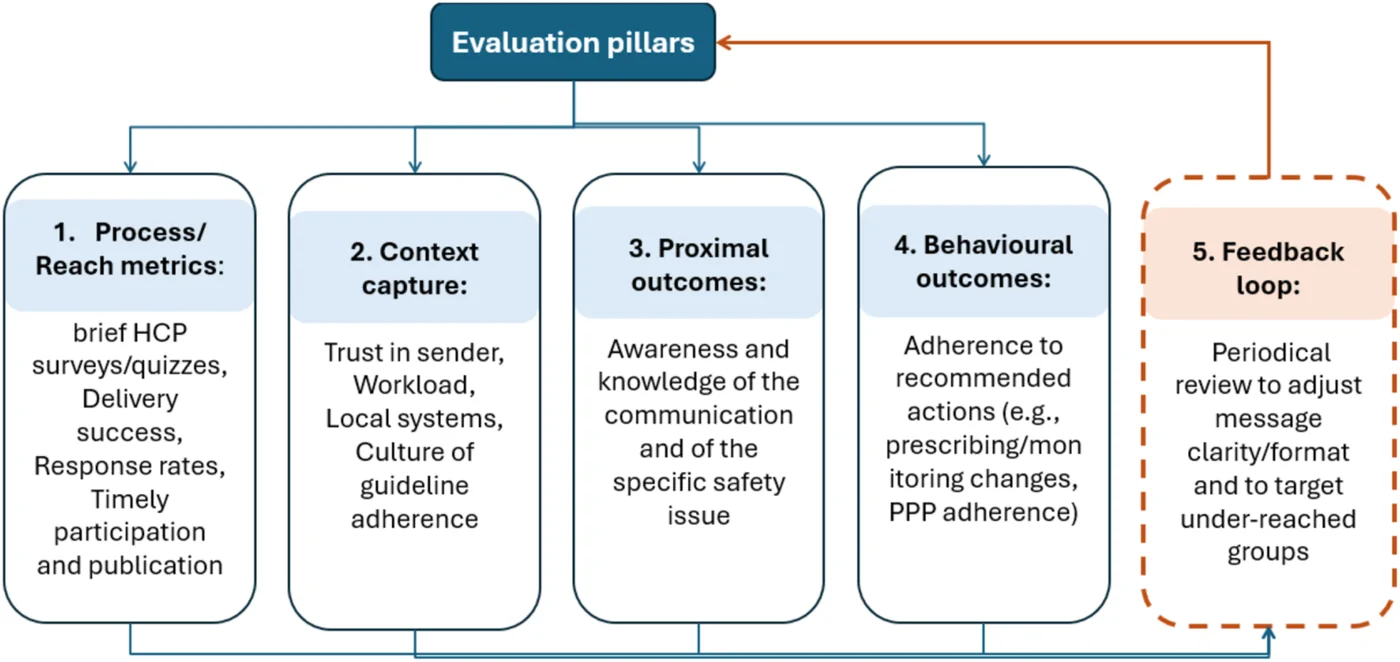
The five pillars of the evaluation process of medicines' risk communications

In another qualitative Danish study (interviews), GPs appeared biased against reading DHPC, for reasons such as considering the sender (pharmaceutical companies) untrustworthy and having pre-existing expectations on the amount of contained information (Møllebæk and Kaae, 2021). GPs also placed the letter more as a legal document rather than risk communication, potentially used for blaming rather than to contain critical information that will help them in their practice (defensive medicine) (Møllebæk and Kaae, 2021). These factors have been attributed to have most probably contributed to disregarding the DHPC by HCPs (Møllebæk and Kaae, 2021; Møllebæk *et al*., 2019). Similarly, to pillars 1 and 2, these findings support the inclusion of proximal and behavioural approaches in order to extract credible outcomes. Assessing HCPs' knowledge and obtaining awareness of the communication and the specific safety issues that they entail, is a critical step for the assessment of its proximal outcomes (Pillar 3, Figure 2). Also, investigating adherence to recommended actions (e.g. prescribing/monitoring changes, PPP adherence) aligns with the behavioural perspective of the evaluation as it falls under the behavioural outcome (Pillar 4, Figure 2). Completing the evaluation process, it is of utmost importance to include a feedback element. A periodical review is vital to adjust the clarity and format of the message and to target under-reached groups (Pillar 5, Figure 2).

Taken together, these studies reveal several recurring patterns and gaps. While surveys consistently show that healthcare professionals value timely safety communication, there is considerable variability in preferred formats and actual uptake (de Vries *et al*., 2017; Hughes *et al*., 2021). Contradictions emerge between reported awareness and observed implementation, with some studies indicating high awareness but limited behavioural change (Hitchings *et al*., 2013), whereas others suggest persistent gaps in knowledge for medicines outside clinicians' immediate specialty (de Vries *et al*., 2018). Methodologically, most studies rely on cross-sectional surveys with small or convenience samples and lack inferential statistical analysis, limiting generalizability. Few investigations incorporate behavioural theory or longitudinal designs to assess sustained impact. These inconsistencies underscore the need for systematic, theory-informed evaluations that move beyond process metrics to examine behavioural outcomes.

It has been well established that risk communications have four major aims: a. to inform and educate, b. to change behaviour and initiate action, c. to alarm recipients with extreme information (disasters, etc.) and d. to enable solutions and collaboration (Covello *et al*., 1986). To situate behavioural factors within established theoretical frameworks, it is helpful to examine how aspects such as trust, perceived relevance, time constraints and habitual practices align with constructs in recognized behaviour-change models. Within the COM-B framework, trust and relevance correspond to reflective motivation, time pressure reflects limitations in opportunity and habitual behaviours relate to automatic motivation. Similarly, the EAST (Easy, Attractive, Social, and Timely) framework underscores the role of defaults, simplicity and social influences, which connect to routine actions and the importance of clear, accessible communication. The Health Belief Model offers comparable concepts: trust aligns with the perceived credibility of cues to action, relevance with perceived benefits and time pressure with perceived barriers. Interpreting the study through these lenses demonstrates that the behavioural themes identified in prior research are consistent with well-established determinants of behaviour in health communication literature.

In the case of disseminating risk communication to HCPs, concerning medicines safety, the aims that are applicable are the information, education, behavioural change (follows after attitude change and persuasion) and action initiation (Covello *et al*., 1986). To achieve these goals, all barriers above will need to be overcome first, as behavioural change cannot happen to a target population that is uninterested and unwilling (Covello *et al*., 1986).

## Conclusion

This conceptual analysis identified four recurring themes across the literature: variability in communication practices, persistent gaps between awareness and implementation, methodological limitations in existing evaluations and the critical role of human factors in shaping prescriber behaviour. These themes informed the development of the proposed five-pillar framework for evaluating medicine risk communications.

National medicines regulatory bodies/authorities are using approved outlets and distribution modes to communicate medicines safety issues in a timely and efficient manner to HCPs. However, literature has identified the need for evaluation of the effectiveness of these risk communication systems. The wide variation in how safety communications are delivered across European countries adds considerable complexity. Although the EMA and national authorities have established structured systems for disseminating safety information, the formats and channels differ substantially between jurisdictions. From a behavioural perspective, this variability affects key factors such as reach, trust, comprehension and implementation – contributing to well-documented gaps between awareness and actual changes in clinical practice. This lack of consistency can weaken the clarity and impact of messages, making it challenging to evaluate their effectiveness in a uniform way.

To answer these questions, risk communications need to be periodically evaluated not only on the dissemination element, but also in knowledge gain, social reach, utilisation and action, aligned with human-factors principles and the established aims of risk communication (inform/educate → attitude/behaviour change → action) (Mayall and Banerjee, 2014). Based on the literature's various results on the acceptance, awareness and preferences of risk communications among HCPs (Mayall and Banerjee, 2014), measures such as:

1. New knowledge and awareness that HCPs gain from the dissemination of risk communications,
2. HCPs' social cognition, which is achieved from the communications' reach,
3. HCPs' utilisation of the risk communications and
4. Their behaviour when action is required

Are key elements for any medicine risk evaluation process. Therefore, the five research-derived pillars of the evaluation process of medicines' risk communications are proposed as a systematic approach in the design of such comprehensive evaluations.

## References

[ref001] Ahlqvist Rastad, J., Andric, A., Baldelli, I., Barrow, P., Bouder, F., Coleman, A.M., Cupelli, A., De Vries, S.T., Escudero, Y., Garcia, J., Haddad, R., Hearn, J., Knudsen, Y., Loughlin, L., Maciá, M.Á., Michan, L., Montero, D., Mol, P., Rodriguez, A., Samdal, H., Sipic, I., Van Der Sar, M. and Wennberg, A. (2016), “Risk communication on medicines: report from the workshop”, in Agency, E.M. (Ed.), Strengthening Collaboration for Operating Pharmacovigilance in Europe (Scope) Joint.

[ref002] Alharbi, A.B., Berrou, I., Umaru, N., Al Hamid, A. and Shebl, N.A. (2023), “Factors influencing the implementation of medicine risk communications by healthcare professionals in clinical practice: a systematic review”, Research in Social and Administrative Pharmacy, Vol. 19 No. 1, pp. 28-56, doi: 10.1016/j.sapharm.2022.07.003. 35989221

[ref003] Back, J., Owens, D. and Benneyworth, R. (2024), “The health services safety investigations body (hssib) and safety management systems: an integrated approach to managing safety in healthcare”, Journal Of Patient Safety And Risk Management, Vol. 29 No. 2, pp. 79-82.

[ref004] Barth, J.H., Misra, S., Aakre, K.M., Langlois, M.R., Watine, J., Twomey, P.J. and Oosterhuis, W.P. (2016), “Why are clinical practice guidelines not followed?”, Clinical Chemistry and Laboratory Medicine, Vol. 54 No. 7, pp. 1133-1139, doi: 10.1515/cclm-2015-0871. 26650076

[ref005] Bhasale, A.L., Sarpatwari, A., De Bruin, M.L., Lexchin, J., Lopert, R., Bahri, P. and Mintzes, B.J. (2021), “Postmarket safety communication for protection of public health: a comparison of regulatory policy in Australia, Canada, the European union, and the United States”, Clinical Pharmacology and Therapeutics, Vol. 109 No. 6, pp. 1424-1442, doi: 10.1002/cpt.2010. 32767557

[ref006] Carlsen, B. and Bringedal, B. (2011), “Attitudes to clinical guidelines–do GPS differ from other medical doctors?”, BMJ Quality and Safety, Vol. 20 No. 2, pp. 158-162, doi: 10.1136/bmjqs.2009.034249. 21209148

[ref007] Chemtob, M.C.C. (2025), “De la transparence au data mining: 14 ans d’évolution de la pharmacovigilance européenne”, Médecine and Droit, Vol. 2025 No. 191, pp. 56-62.

[ref008] Covello, V.T., Slovic, P. and Von Winterfeldt, D. (1986), “Risk communication: a review of the literature”, Risk Abstracts, Vol. 3, pp. 171-182.

[ref009] De Vries, S.T., Van Der Sar, M.J., Cupelli, A., Baldelli, I., Coleman, A.M., Montero, D., Šipić, I., Andrić, A., Wennberg, A., Ahlqvist-Rastad, J., Denig, P. and Mol, P.G.M. (2017), “Communication on safety of medicines in Europe: current practices and general practitioners' awareness and preferences”, Drug Safety, Vol. 40 No. 8, pp. 729-742, doi: 10.1007/s40264-017-0535-0. PMC5519651 28540672

[ref010] De Vries, S.T., Van Der Sar, M.J., Coleman, A.M., Escudero, Y., Rodríguez Pascual, A., Maciá Martínez, M.-Á., Cupelli, A., Baldelli, I., Šipić, I., Andrić, A., Michan, L., Denig, P. and Mol, P.G.M. (2018), “Safety communication tools and healthcare professionals' awareness of specific drug safety issues in Europe: a survey study”, Drug Safety, Vol. 41 No. 7, pp. 713-724, doi: 10.1007/s40264-018-0643-5. PMC5990574 29500798

[ref011] Durand, M., Castelli, C., Roux-Marson, C., Kinowski, J.-M. and Leguelinel-Blache, G. (2024), “Evaluating the costs of adverse drug events in hospitalized patients: a systematic review”, Health Economics Review, Vol. 14 No. 1, p. 11, doi: 10.1186/s13561-024-00481-y. PMC10851489 38329561

[ref012] Heinström, J. (2003), “Five personality dimensions and their influence on information behaviour”, Information Research, Vol. 9, pp. 9-1.

[ref013] Hitchings, A.W., Wood, D.M. and Dargan, P.I. (2013), “Dissemination and uptake of a new treatment pathway for paracetamol poisoning in the UK: a survey of healthcare professionals”, British Journal of Clinical Pharmacology, Vol. 76 No. 6, pp. 946-950, doi: 10.1111/bcp.12111. PMC3845318 23488574

[ref014] Hughes, J.E., Buckley, N., Looney, Y., Kirwan, G., Curran, S., Doherty, C.P., Mullooly, M. and Bennett, K.E. (2021), “Awareness, knowledge and practice of healthcare professionals following implementation of a pregnancy prevention program for sodium valproate in Ireland: a multi-stakeholder cross-sectional study”, Expert Opinion on Drug Safety, Vol. 20 No. 8, pp. 965-977, doi: 10.1080/14740338.2021.1933429. 34080507

[ref015] Kastner, M., Bhattacharyya, O., Hayden, L., Makarski, J., Estey, E., Durocher, L., Chatterjee, A., Perrier, L., Graham, I.D., Straus, S.E., Zwarenstein, M. and Brouwers, M. (2015), “Guideline uptake is influenced by six implementability domains for creating and communicating guidelines: a realist review”, Journal of Clinical Epidemiology, Vol. 68 No. 5, pp. 498-509, doi: 10.1016/j.jclinepi.2014.12.013. 25684154

[ref016] Khalaf, Z., Saglani, S. and Bloom, C.I. (2024), “Implementation and effectiveness of guide line recommended clinical activities for children with asthma: population-based cohort”, Chest, Vol. 167 No. 3, pp. 665-674. 10.1016/j.chest.2024.10.036 PMC11882771 39515516

[ref017] Kil, Y., Graham, M. and Chatzi, A.V. (2024), “Examination of personality types as predictors of safety attitudes/behaviours, in Support of Enhancing Safety in Healthcare: A Scoping Review”, International Journal of Health Governance.

[ref018] Lugtenberg, M., Burgers, J.S., Besters, C.F., Han, D. and Westert, G.P. (2011), “Perceived barriers to guideline adherence: a survey among general practitioners”, BMC Family Practice, Vol. 12, pp. 1-9, doi: 10.1186/1471-2296-12-98. PMC3197492 21939542

[ref019] Mayall, S. and Banerjee, A. (2014), “Evaluating the effectiveness of risk minimisation”, in Therapeutic Risk Management of Medicines, Elsevier.

[ref020] Medicines and Healthcare Products Regulatory Authority (2024), “Mhra strategy for improving safety communications 2024-2027”, in Authority, M.A.H.P.R. (Ed.), United Kingdom: Medicines and Healthcare Products Regulatory Authority.

[ref021] Møllebæk, M. and Kaae, S. (2021), “Why do general practitioners disregard Direct to healthcare professional communication? A user‐oriented evaluation to improve drug safety communication”, Basic and Clinical Pharmacology and Toxicology, Vol. 128 No. 3, pp. 463-471, doi: 10.1111/bcpt.13516. 33043558

[ref022] Møllebæk, M. and Kaae, S. (2022), “Are drug safety advisories compatible with physicians' information behaviour? Semi-structured interviews with general practitioners about direct to healthcare professional communication”, European Journal Of Health Communication, Vol. 3, pp. 1-17, doi: 10.47368/ejhc.2022.301.

[ref023] Møllebæk, M., Kaae, S., De Bruin, M.L., Callréus, T., Jossan, S. and Hallgreen, C.E. (2019), “The effectiveness of direct to healthcare professional communication – a systematic review of communication factor studies”, Research in Social and Administrative Pharmacy, Vol. 15 No. 5, pp. 475-482, doi: 10.1016/j.sapharm.2018.06.015. 31130181

[ref024] Nyeland, M.E., Laursen, M.V. and Callréus, T. (2017), “Evaluating the effectiveness of risk minimisation measures: the application of a conceptual framework to Danish real‐world dabigatran data”, Pharmacoepidemiology and Drug Safety, Vol. 26 No. 6, pp. 607-614, doi: 10.1002/pds.4203. 28397317

[ref025] O'connor, P. and O'dea, A. (2021), An Introduction to Human Factors for Healthcare Workers, Ireland.

[ref026] Perry, L.T., Mohammad, A., Hooimeyer, A., Mcewin, E.J. and Mintzes, B. (2025), “How does the content and dissemination of communications on the risks of medicines affect prescriber awareness, knowledge, and behaviour: a systematic review”, Drug Safety, Vol. 49, pp. 1-27, doi: 10.1007/s40264-025-01588-9. PMC12804208 40758211

[ref027] Santoro, A., Genov, G., Spooner, A., Raine, J. and Arlett, P. (2017), “Promoting and protecting public health: how the European Union pharmacovigilance system works”, Drug Safety, Vol. 40 No. 10, pp. 855-869, doi: 10.1007/s40264-017-0572-8. PMC5606958 28735357

[ref028] Vaghi, A., Incalzi, R.A., Barbaglia, S., Bilò, M.B., Bini, F., Carone, M., Cecchi, L., Chetta, A.A., Comel, A.C., De Michele, F., Insalaco, G., Musarra, A., Pomponio, G., Spanevello, A., Tognella, S., Vatrella, A., Zuccatosta, L. and Micheletto, C. (2025), “Expert opinion on gray areas in asthma management: a lesson from the innovative project ‘revolution in asthma’ of the Italian thoracic society (Aipo‐Its)”, Clinical and Translational Allergy, Vol. 15 No. 2, E70037, doi: 10.1002/clt2.70037. PMC11807766 39924642

[ref029] Weatherburn, C.J., Guthrie, B., Dreischulte, T. and Morales, D.R. (2020), “Impact of medicines regulatory risk communications in the UK on prescribing and clinical outcomes: systematic review, time series analysis and meta‐analysis”, British Journal of Clinical Pharmacology, Vol. 86 No. 4, pp. 698-710, doi: 10.1111/bcp.14104. PMC7098864 31465123

[ref030] Wiener-Ogilvie, S., Pinnock, H., Huby, G., Sheikh, A., Partridge, M.R. and Gillies, J. (2007), “Do practices comply with key recommendations of the British asthma guideline? If not”, Why Not? Primary Care Respiratory Journal, Vol. 16 No. 6, pp. 369-377, doi: 10.3132/pcrj.2007.00074. PMC6634244 18026674

[ref031] World Health Organization (2011), Patient Safety Curriculum Guide: Multi-Professional Edition, Who Press, Switzerland.

[ref032] World Health Organization (2019), “Patient safety”, [Online], available at: https://Www.Who.Int/News-Room/Facts-In-Pictures/Detail/Patient-Safety (accessed 6 December 2022).

[ref033] World Health Organization (2023), “Patient safety”, [Online], available at: https://Www.Who.Int/News-Room/Fact-Sheets/Detail/Patient-Safety (accessed 15 September 2023).

